# Comparative chloroplast genome analyses of cultivated spinach and two wild progenitors shed light on the phylogenetic relationships and variation

**DOI:** 10.1038/s41598-022-04918-4

**Published:** 2022-01-17

**Authors:** Hongbing She, Zhiyuan Liu, Zhaosheng Xu, Helong Zhang, Feng Cheng, Jian Wu, Xiaowu Wang, Wei Qian

**Affiliations:** Institute of Vegetables and Flowers, Chinese Academy of Agricultural Sciences, Beijing, 100081 China

**Keywords:** Phylogenetics, Population genetics

## Abstract

*Spinacia* is a genus of important leafy vegetable crops worldwide and includes cultivated *Spinacia oleracea* and two wild progenitors, *Spinacia turkestanica* and *Spinacia tetrandra*. However, the chloroplast genomes of the two wild progenitors remain unpublished, limiting our knowledge of chloroplast genome evolution among these three *Spinacia* species. Here, we reported the complete chloroplast genomes of *S. oleracea*, *S. turkestanica*, and *S. tetrandra* obtained via Illumina sequencing. The three chloroplast genomes exhibited a typical quadripartite structure and were 150,739, 150,747, and 150,680 bp in size, respectively. Only three variants were identified between *S. oleracea* and *S. turkestanica*, whereas 690 variants were obtained between *S. oleracea* and *S. tetrandra*, strongly demonstrating the close relationship between *S. turkestanica* and *S. oleracea*. This was further supported by phylogenetic analysis. We reported a comprehensive variant dataset including 503 SNPs and 83 Indels using 85 *Spinacia* accessions containing 61 *S. oleracea*, 16 *S. turkestanica*, and eight *S. tetrandra* accessions. Thirteen *S. oleracea* accessions were derived through introgression from *S. turkestanica* that acts as the maternal parent. Together, these results provide a valuable resource for spinach breeding programs and improve our understanding of the phylogenetic relationships within Amaranthaceae.

## Introduction

Genome sequences are frequently used for elucidating evolutionary processes, genetic diversity, interest-trait mapping, and phylogenic relationships^1–3^. In plants, the chloroplast, mitochondrion, and nucleus contribute genetic information to offspring^4^. The chloroplast genome is small in size, has single maternal inheritance, has a low nucleotide substitution rate, and has a highly conserved gene order and gene content in comparison to the nuclear genome^5^. Therefore, the chloroplast genome is considered an ideal model for investigating diversity and evolution.

Chloroplasts, which are unique to algae and plants, are cell organelles that are involved in photosynthesis, the process of converting light to energy^6,7^. Shinozaki et al. ^8^ first assembled the complete tobacco chloroplast genome. To date, more than 6000 complete chloroplast genomes have been recorded by the GenBank database (https://www.ncbi.nlm.nih.gov/genbank/). Most chloroplast genomes have a double-stranded, circular, typical quadripartite structure with a pair of inverted repeats (IRs) separated by a large single copy (LSC) region and a small single-copy (SCC) region^9^. Generally, chloroplast genomes have a DNA length of 120–160 Kb, about 79 protein-coding genes, 30 transfer RNAs (tRNAs), and four ribosomal RNAs (rRNAs)^10^. The variation in size of chloroplast genomes mainly originates from IR expansion/contraction or loss, which has played a vital role in evolution^11,12^. For example, the chloroplast genomes of some algae and legumes do not contain an IR region, resulting in a shorter chloroplast genome length^4^.

Spinach (*Spinacia oleracea* L.) is mostly a dioecious species belonging to the family Amaranthaceae^13–15^. This family is composed of approximately 175 genera and more than 2500 species and is distributed nearly worldwide. A number of species, including spinach, beets, and quinoa, are important food crops. Two wild spinach, species, *S. turkestanica* Iljin and *S. tetrandra* Stev., are distributed in different regions—*S. turkestanica* is located in Central and Southern Asia, while *S. tetrandra* occurs in the Middle East and Transcaucasia^16,17^. Previous investigations demonstrated that *S. turkestanica* was more closely related to the cultivated *S. oleracea* than *S. tetrandra*^13,16,18^. These wild species have been proven to be a valuable genetic resource for improving the quality of spinach varieties, including regarding disease and pest resistance^19,20^. However, the genetic resource collection of wild species is limited thus far, and consequently, the genetic structure of spinach germplasms is still largely unclear. Therefore, increasing the collection of valuable genetic sources and exploring the genetic diversity and phylogenetic relationships of spinach germplasms should help inform elite germplasm utilization and improve spinach breeding programs. Furthermore, chloroplast genome sequences in *Spinacia* have remained limited to date, as only the *S. oleracea* chloroplast genome has been reported^21^.

In this study, we reported three complete chloroplast genomes of *S. tetrandra, S. turkestanica,* and *S. oleracea* using next-generation sequencing technology. By comparing the three chloroplast genomes to each other and to previously reported chloroplast genomes from Amaranthaceae, we specifically aimed to (1) elucidate the genetic diversity and conservation of the chloroplast genomes in *Spinacia*; (2) identify and develop optimized markers for discriminating different spinach species; and (3) assess the phylogenetic relationships within Amaranthaceae using chloroplast genome sequences.

## Results

### Characterization of chloroplast genomes in *Spinacia* species

We generated a total of 400,673,634, 377,024,012, and 335,160,130 paired-end (150 bp) clean reads for *S. oleracea*, *S. turkestanica*, and *S. tetrandra*, respectively. Among these clean reads, 0.93% (3,763,621), 1.24% (4,712,787), and 1.51% (5,078,425) of the clean reads mapped on the reference chloroplast genome sequence of *S. oleracea* (GenBank Accession Number: NC_002202.1) were used for the de novo assembly of *S. oleracea*, *S. turkestanica*, and *S. tetrandra*, respectively. Finally, we obtained three complete chloroplast genomes for these *Spinacia* species. Detailed information on the chloroplast genomes of the three *Spinacia* species is provided in Table 1.

**Table 1:** Summary of the three *Spinacia* species chloroplast genomes.

Category	*Spinacia oleracea*	*Spinacia turkestanica*	*Spinacia tetrandra*
Genome size (bp)	150,739	150,747	150,680
LSC size (bp)	82,725	82,733	82,525
SSC size (bp)	17,868	17,868	17,959
IR size (bp)	25,073	25,073	25,098
Number of genes	143	143	143
Protein-Coding genes	98	98	98
tRNA genes	37	37	37
rRNA genes	8	8	8
GC content (%)	36.81	36.81	36.79
GC content in LSC (%)	34.79	34.79	34.75
GC content in SSC (%)	29.79	29.79	29.83
GC content in IR (%)	42.65	42.65	42.63
Total clean reads	400,673,634	377,024,012	335,160,130
Mapped clean reads	3,763,621	4,712,787	5,078,425

**Figure 1:**
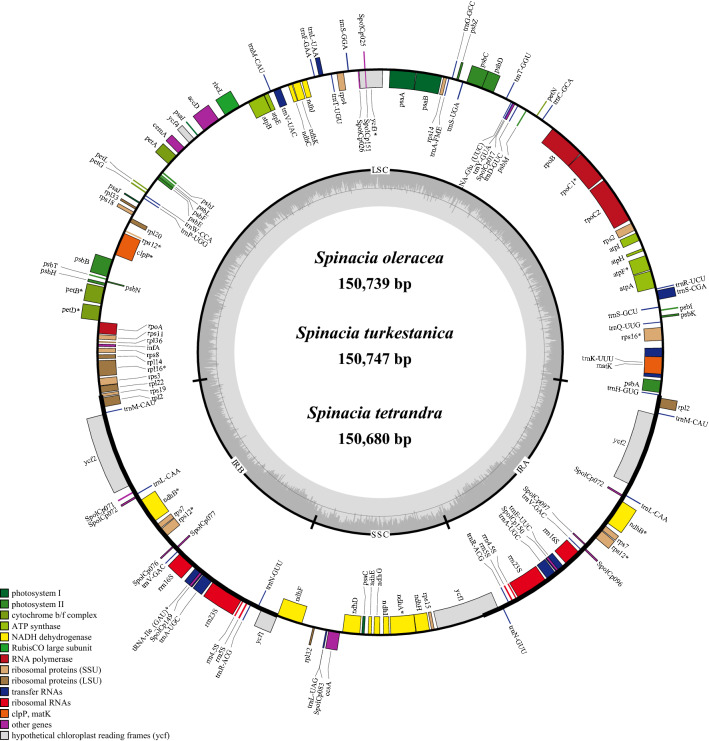
The chloroplast genome maps of the three *Spinacia* species. Genes shown inside the circle are transcribed clockwise, and those outside the circle are transcribed counterclockwise. Genes belonging to different functional groups are color-coded.

The chloroplast genomes of *S. oleracea*, *S. turkestanica*, and *S. tetrandra* shared a typical quadripartite structure containing two copies of an IR region that separate the LSC and SSC regions (Table 1 and Fig. 1). Specifically, the LSC region was from 82,525 to 82,733 bp, which was longer than the SSC (17,868–17,959 bp) and IR regions (25,073–25,098 bp). Furthermore, the overall GC content of the three *Spinacia* chloroplast genomes was approximately 36%. Interestingly, the IR region exhibited a higher GC content (~ 42%) than the LSC (~ 34%) and SSC regions (29%).

The three chloroplast genomes encompassed an identical set of 143 genes, including 98 protein-coding genes, 37 tRNA genes, and eight rRNA genes (Table 1). The gene content, order, and orientation in the three *Spinacia* species were similar (Fig. 1). All eight rRNA were located in the IRs, 23 tRNA genes existed in the single-copy region, and the others were found in the IRs. Among the 12 intron-containing genes, a total of nine genes (*rps16*, *atpF*, *rpoC1*, *petB*, *petD*, *rpl1p6*, *ndhB*, *tRNA-Ile*, and *ndhA*) had one intron while three genes (*ycf3*, *clpP*, and *rps12*) possessed two introns (Table S1). Significantly, rps12 was a trans-spliced gene with one of its exons (5’_end) located in the LSC regions while the other (3’_end) existed in the IR regions. The chloroplast genome sequences were deposited in GenBank (accession numbers: MZ516907 for *S. oleracea*, MZ516906 for *S. turkestanica*, and MZ569012 for *S. tetrandra*).

Shrinkage and expansion of the IR regions might account for the different sizes of the chloroplast genomes. Thus, we compared the IR border position and its adjacent genes between the three *Spinacia* chloroplast genomes (Fig. S1). The *rps19*, *ndhF*, *ycf1*, *rpl2*, and *trnH-GUG* genes were positioned at the junctions of the LSC/IRB, IRB/SSC, SSC/IRA, and IRA/LLC regions, respectively. Interestingly, the border position in *S. oleracea* was the same as that in *S. turkestanica*, which implied no IR expansion or contraction. Additionally, only two genes, *ycf1* and *rpl2*, exhibited different boundary regions among the three *Spinacia* chloroplast genomes. Specifically, ycf1 was 4057 bp in the SSC regions of *S. oleracea* and *S. turkestanica* but 4063 bp in the SSC region of *S. tetrandra*. The gene *rpl2* was 819 bp size in *S. oleracea* and *S. turkestanica* and 825 bp in *S. tetrandra*. Altogether, no significant shrinkage/expansion of the IR regions, especially between *S. oleracea* and *S. turkestanica*, was detected among the *Spinacia* species. Therefore, we propose that the shrinkage/expansion of the IR regions is not the main reason for the different sizes of the chloroplast genomes in the study.

### Phylogenetic analysis and *Spinacia* chloroplast genome evolution

**Figure 2:**
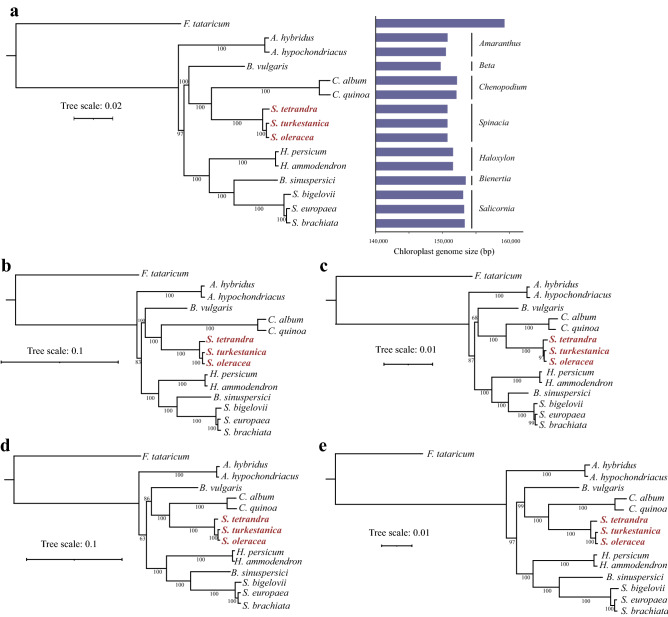
Phylogenetic trees of the 14 Amaranthaceae plants based on chloroplast genomes and the variation in their chloroplast genome size. The phylogenetic tree was constructed using the (**a**) complete chloroplast genome data, (**b**) LSC region, **(c)** IR region, (**d**) SSC region, and (**e**) protein sequences, with *F. tataricum* as an outgroup. Numbers near branches are bootstrap values.

To explore the phylogenetic position of *Spinacia* and further clarify its evolutionary relationships with other species from the Amaranthaceae family, 14 Amaranthaceae species were selected for phylogenetic tree construction with *Fagopyrum tataricum* (Polygonaceae) as an outgroup (Table S2). We utilized different data, including the complete chloroplast genome, LSC, IR, SSC, and protein sequences to construct the phylogenetic trees (Fig. 2). All of the phylogenetic trees had the same topology with high bootstrap support. Specifically, the 14 species from the Amaranthaceae family grouped into one cluster, which was further divided into seven clusters corresponding to different genera. Furthermore, *Amaranthus* was placed in the most basal clade among the Amaranthaceae species, and then *Beta*, *Chenopodium* and *Spinacia* formed a sister clade to *Haloxylon*, *Bienertia* and *Salicornia*. Additionally, the three *Spinacia* species were more closely related to *Chenopodium* than *B. vulgaris*, with 100% bootstrap support values, which is consistent with a previous study using single-copy genes from the genome^13,22^. In the resulting phylogenies, we confirmed, based on chloroplast genome that *S. turkestanica* exhibited a closer relationship with *S. oleracea* than with *S. tetrandra*, and the chloroplast genomes were conserved, as the phylogenetic trees of the quadripartite structures shared the same topology.

### Variant and population genetic analyses across *Spinacia* species

**Figure 3:**
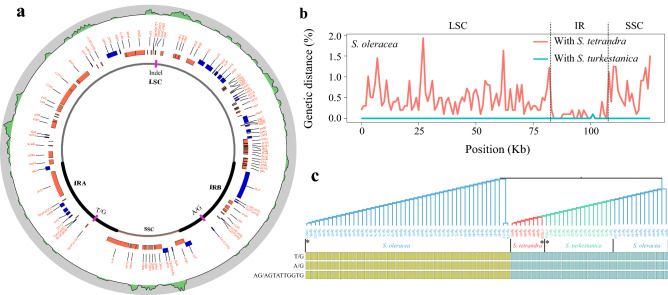
Variability of the three *Spinacia* species based on the chloroplast genome. (**a**) The purple lines in the innermost circle correspond to variants between *S. turkestanica* and *S. oleracea*. The green region in the outermost circle corresponds to variants between *S. tetrandra* and *S. oleracea* with a 1-Kb window and 100-bp steps. Genes represented by blue rectangles are on the positive strand while genes represented by red rectangles are on the negative strand. (**b**) Genetic distance between *S. oleracea* and the wild relative chloroplast assemblies of *S. tetrandra* (red) and *S. turkestanica* (blue). (**c**) The phylogenetic tree was constructed based on three variants between *S. turkestanica* and *S. oleracea* using 85 *Spinacia* accessions, including eight *S. tetrandra*, 16 *S. turkestanica*, and 61 *S. oleracea* individuals. Yellow rectangles indicate that the homozygous genotype in the accession was consistent with the reference chloroplast genome, whereas blue rectangles indicate that the homozygous genotype differed from the reference chloroplast genome.

To infer the variation between the cultivated and two wild progenitors, we aligned the complete chloroplast genome sequences of *S. turkestanica* and *S. tetrandra* against the *S. oleracea* chloroplast genome sequence. A total of three and 690 variants were identified in this comparison (Table 2). The three variants within *S. oleracea* and *S. turkestanica* were also detected between *S. oleracea* and *S. tetrandra*, and thus a total of 690 non-redundant variants, including 559 single nucleotide polymorphisms (SNPs) and 131 insertion-deletions (Indels), were obtained among *S. oleracea*, *S. turkestanica*, and *S. tetrandra*. Among the 690 non-redundant variants, the average variant density in the LSC and SSC regions was 6.3 variants per kb, whereas this value was only 0.9 variants per kb in the IR regions (Fig. 3a and Table 2), revealing that the IR regions were more conserved than the single-copy regions, which was further supported by the genetic distance between *S. oleracea* and its two wild relatives (Fig. 3b).

**Table 2:** Summary of the variation between *S. oleracea* and the two wild progenitors.

Items	*Spinacia turkestanica*		*Spinacia tetrandra*	
	SNP	Indel	SNP	Indel
Numbers of variant	2	1	559	131
LSC	0	1	403	93
IR	2	0	38	10
SSC	0	0	118	28
Coding	0	0	230	10
Synonymous	0	–	138	–
Nonsynonymous	0	–	92	–
Intron	2	0	67	41
Intergenic	0	1	262	80

Furthermore, analysis of the distribution of the 690 variants revealed that 342 (49%) of them were situated in intergenic regions, 108 (15%) were located in intron regions, and 240 (34%) were positioned in coding regions (Table 2). Two hundred and thirty SNPs out of the 240 variants within the coding regions were further divided into 92 nonsynonymous and 138 synonymous SNPs, which were located in 42 genes (Fig. S2). Among these synonymous SNPs, 61 (44%) SNPs were enriched on the *ndhF*, *psaA*, *rpoB*, *ropC2*, *rbcL*, and *ycf1* genes. Only three out of the 10 Indels within the coding regions were predicted to result in frameshifts of the *matK* and *rpl22* genes (Fig. S2).

**Figure 4:**
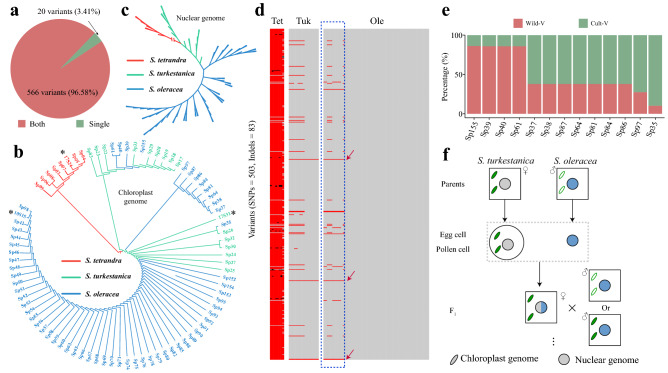
Population genetic analysis of the 85 *Spinacia* species. (**a**) Comparison of the variant data detected by the 82 resequencing accessions and the three complete chloroplast genomes. ‘Both’ indicates that the SNPs detected by 82 resequencing accessions were present in the SNP dataset from the three chloroplast genomes, whereas ‘Single’ indicates that the SNPs were specifically detected in the 82 resequencing accessions. A neighbor-joining tree of 85 *Spinacia* accession using the spinach variants’ (**b**) chloroplast genome and (**c**) nuclear genome. The individuals with asterisks are used to assembled in the study. (**d**) Variants in *S. oleracea*, *S. turkestancia*, and *S. tetrandra*. Gray lines indicate reference (*S. oleracea*) homozygous genotype, while red and black lines represent alternative homozygous and heterozygous genotype, respectively. The samples surrounded with blue dotted lines are the 13 *S. oleracea* clustered with *S. turkestanica* in (**b**). The red arrows mean the three conserved variants between *S. oleracea* and wild progenitors in Fig. 2c. Tet, *S. tetrandra*; Tuk, *S. turkestanica*; Ole, *S. oleracea*. (**e**) The percentage of variants (from wild or cultivar) in 29 variants existing in 13 *S. oleracea* clustered with *S. turkestanica* in Fig. D. Wild-V indicates the variants from wild species, while Cult-V represents the variants from cultivar species. (**f**) The inheritance patterns of the 13 *S. oleracea*.

Interestingly, only three variants were identified between *S. oleracea* and *S. turkestanica*. To validate that the three variants specifically existed in *S. oleracea*, we constructed a phylogenetic tree based on the three variants using the 85 spinach accessions including eight *S. tetrandra*, 16 *S. turkestanica*, and 61 *S. oleracea* individuals^13^ (Fig. 3c). The result showed that almost all of *S. oleracea* and the two wild relatives could be separated into two distinct clades with different genotypes of the three variants, whereas there were still 13 *S. oleracea* individuals in the same clade as the wild progenitors (Fig. 3c). Furthermore, we detected 503 SNPs and 83 indels using the 85 spinach accessions. A total of 566 (96.58%) of these variants were shared in the 690 variants obtained by global sequence alignment of the three chloroplast genomes (Fig. 4a). Similarly, a neighbor-joining phylogeny based on these variants still showed that the 13 *S. oleracea* and *S. turkestanica* accessions clustered together (Fig. 4b). However, all of the *S. oleracea* accessions clustered together based on the 1,084,637 variants identified from spinach nuclear genome^23^ (Fig. 4c). Meanwhile, we noticed that the 13 *S. oleracea* accessions exactly include 29 variants from *S. turkestanica* (Fig. 4d). Of these 29 variants, more than 86% variants were existed in Sp155, Sp39, Sp40, and Sp61, while Sp35 encompassed the lowest number of variants from *S. turkestanica* (Fig. 4e), which was consistent with the phylogenetic tree (Fig. 4b).

Importantly, chloroplasts of most species are inherited from the female parent^24^. Therefore, we propose that the 13 *S. oleracea* have cross-pollinated with *S. turkestanica*, with the latter having been the female parent, followed by continuously cross or/and backcross with paternal parents of *S. oleracea* (Fig. 4f). Consequently, the 13 *S. oleracea* accessions bear a similar chloroplast genome to *S. turkestanica* and a similar nuclear genome to *S. oleracea*. This provided an effective approach for us to create new spinach germplasm.

### Repeat sequence analysis

**Figure 5:**
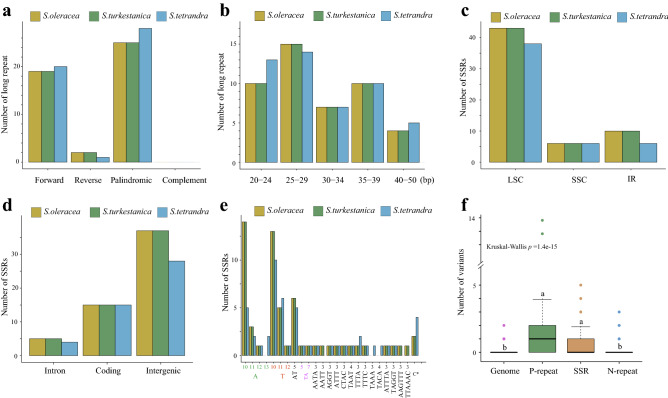
Repeat sequences in the chloroplast genomes of three *Spinacia* species. (**a**) Number of different long-repeat types and (**b**) length distribution in the *Spinacia* species. **(c)** Distribution of SSRs in the LSC, SSC, and IR regions, as well as (**d**) intron, coding, and intergenic regions. (**e**) Analysis of SSRs for different lengths of repeat (bp) and repeated sequences. (**f**) Comparison of variant (SNPs and Indels) numbers between the repeat regions and equivalently sized regions randomly selected from the chloroplast genome. The repeat regions indicate upstream and downstream 50-bp repeats. The genome represents the 50 regions randomly sampled from the chloroplast genome (including upstream and downstream 50-bp repeats). The P-repeat refers to polymorphic long-repeat regions, whereas N-repeat refers to non-polymorphic long-repeat regions. The different letters on the top of the boxplot indicate significant differences (Kruskal–Wallis test, *P* < 1.4e−15).

A total of 46, 46, and 49 pairs of repeats (≥ 20 bp), termed long repeats in this study, were identified for *S. oleracea*, *S. turkestanica*, and *S. tetrandra*, respectively, using the program REPuter^25^ (Table S3). Specifically, 19, 19, and 20 forward repeats, two, two, and one reverse repeats, 25, 25, and 28 palindromic repeats, and no complementary repeats were obtained in *S. oleracea*, *S. turkestanica*, and *S. tetrandra*, respectively (Fig. 5a). The length distribution of the long repeat sequences was mainly 25–29 bp and rarely 40–45 bp among the three *Spinacia* species (Fig. 5b). Significantly, the long repeat sequences of *S. oleracea* and *S. turkestanica* were identical, with only three variants found between them (Fig. 3a). Importantly, none of the long repeats located in the three variants were identified. The majority of long repeats in the three *Spinacia* species were situated in intergenic regions, particularly, between rrn4.5 and rrn5 (Table S3). The ycf3 intron and ycf2 coding region also exhibited multiple nested long repeats (Table S3). Eleven pairs of polymorphic long-repeat sequences were identified, seven of which were *S. tetrandra*-specific repeats, and the remaining were *S. oleracea*/*S. turkestanica*-specific repeats (Fig. S3a).

Additionally, we also identified 59, 59, and 50 simple sequence repeats (SSRs) in *S. oleracea*, *S. turkestanica*, and *S. tetrandra*, respectively (Table S4). Similar to the observations in the long repeat sequences, *S. oleracea* shared the same SSRs as *S. turkestanica*, supporting the closer relationship between them. Among these SSRs, the vast majority were enriched in the LSC region, and *S. tetrandra* shared less SSRs than *S. oleracea*/*S. turkestanica* in the LSC and IR regions (Fig. 5c). These SSRs from the three *Spinacia* species were mainly located in intergenic regions (56–62%), followed by coding regions (25–30%), and only five and four SSRs were detected in the intron regions for *S. oleracea*/*S. turkestanica* and *S. tetrandra*, respectively (Fig. 5d). Single-nucleotide SSRs accounted 62.7%, 62.7%, and 54% of the total number of SSRs in *S. oleracea*, *S. turkestanica*, and *S. tetrandra*, respectively (Fig. 5e). There were multiple polymorphic SSRs between *S. oleracea*/*S. turkestanica* and *S. tetrandra*. For example, (A)_13_, (TAAA)_3_, and (TACA)_4_ SSRs were shared in *S. tetrandra*, but not in *S. oleracea* and *S. turkestanica*. In contrast, (AATT)_3_, (AAGTTT)_3_, and (TTAAAC)_3_ were specific to *S. oleracea* and *S. turkestanica* (Fig. 5e).

To explore the relationship between variants (SNPs and Indels) and these repeats among the three *Spinacia* chloroplast genomes, we compared the variation levels within the polymorphic, non-polymorphic long repeats, SSRs, and their flanking regions with equivalently sized regions randomly selected from the chloroplast genome, which revealed that the polymorphic long repeats, SSRs, and their flanking regions exhibited multiple variants, whereas the non-polymorphic long repeats and their flanking regions did not (Fig. 5f). Taken together, these findings suggested that the polymorphic long repeats and SSRs were highly variable and have been essential in spinach chloroplast genome evolution. Furthermore, variants could be regarded as important factors for generating polymorphic repeats. For example, the 11 polymorphic long repeats were generated due to the repeat sequences being destroyed by SNPs or Indels (Figs. S3b, c). A phylogenetic tree was constructed using 85 *Spinacia* accessions including eight *S. tetrandra* and 77 *S. oleracea*/*S. turkestanica* accessions based on the five variants identified from the polymorphic repeats (Fig. S3d). The phylogenetic tree showed that seven *S. tetrandra* and 75 *S. oleracea*/*S. turkestanica* accessions could obviously be divided into two clades based on the five variants, thus revealing that these variants or polymorphic repeats have excellent value for molecular marker investigation and spinach breeding programs.

### Rates of synonymous and non-synonymous substitutions

**Figure 6:**
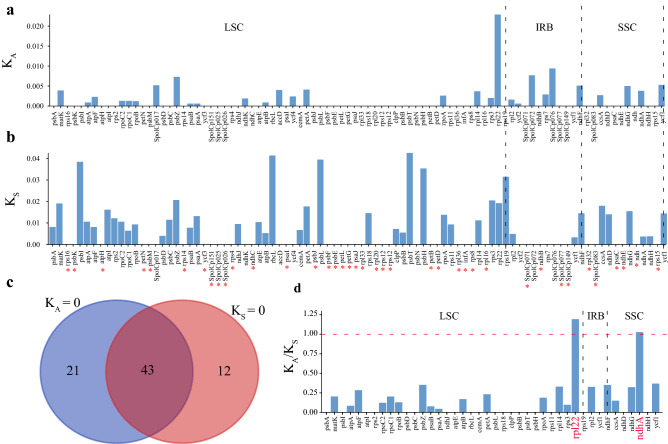
Non-synonymous (K_A_), synonymous (K_S_), and K_A_/K_S_ substitution values of *S. tetrandra*. (**a**) K_A_, (**b**) K_S_, and (**d**) K_A_/K_S_ substitution values. (**c**) Venn diagram of protein-coding genes with K_A_ = 0 and K_S_ = 0. The genes highlighted in red were under positive selection during the *Spinacia* evolution.

Non-synonymous (K_A_), synonymous (K_S_) nucleotide substitution rates, and the ratio of K_A_/K_S_ are widely defined as indicators of the selective pressures on genes during evolution. To examine the molecular evolution of chloroplast protein-coding genes in the *Spinacia* species, we calculated the K_A_, K_S_, and K_A_/K_S_ values of 98 chloroplast protein-coding genes from *S. turkestanica* and *S. tetrandra*, with *S. oleracea* as a reference (Fig. 6). The K_A_ and K_S_ values of *S. turkestanica* were zero, strongly suggesting a closer relationship between *S. turkestanica* and *S. oleracea,* as only three variants (not in the coding region) existed (Table S5). However, the K_A_ values of *S. tetrandra* ranged from 0.0000 to 0.0229 (*rpl22*), and the K_S_ values ranged from 0.0000 to 0.0426 (*psbT*) (Figs. 6a, b). Otherwise, 64 (65%) and 55 (56%) genes did not show K_A_ and K_S_ rate changes in *S. tetrandra*, respectively. Among these genes, 43 genes shared zero values for both K_A_ and K_S_, suggesting that these genes were highly conserved during *Spinacia* evolution (Fig. 6c and Table S5). Twenty-three out of 98 chloroplast protein-coding genes exhibited K_A_/K_S_ values greater than 0, and only the *rpl22* and *ndhA* genes, located in LSC and SSC, respectively, were under positive selection (Fig. 6d). The *rpl22* and *ndhA* genes are related to photosynthesis and transcription or translation, respectively.

## Discussion

Chloroplast genomes are known to be highly conserved in both gene order and gene content^26^. The three *Spinacia* chloroplast genomes shared good collinearity and the same numbers of genes (Table 1 and Fig. 1). Due to their conserved characteristic, chloroplast genomes are valuable for evolutionary analysis^5,27^. Earlier phylogenetic analyses utilized partial chloroplast sequences, such as LSC, SSC, IRs, or CDS sequences. However, these sequences might not provide complete information for each species, and thus the use of complete chloroplast genome sequences is regarded as a more effective approach for deciphering phylogenetic relationships, especially for closely related taxa^6,7^. In this study, we constructed phylogenetic trees of 14 Amaranthaceae species based on complete chloroplast genome sequences, LSC regions, IR regions, SSC regions, and protein sequences (Fig. 2 and Table S2). Remarkably, the phylogenetic trees based on the partial chloroplast sequences and complete chloroplast genome exhibited the same topology with high bootstrap support, strongly indicating the conserved nature of the chloroplast genomes of the 14 Amaranthaceae species and further demonstrating that the phylogenetic tree was highly reliable (Fig. 2). Furthermore, both the phylogenetic tree and genetic distances between *S. oleracea* and the two wild relatives revealed that *S. turkestanica* shared a closer relationship with *S. oleracea* than *S. tetrandra* did, which is consistent with previous studies^13,16,23^. To the best of our knowledge, this is the first comprehensive phylogenetic analysis of Amaranthaceae species using different information and is thus a valuable resource for deciphering the evolutionary relationships of Amaranthaceae.

In general, the IR regions are believed to be the most conserved of the chloroplast genome^28–30^. The distribution of the variants on the chloroplast genome and the genetic distances between *S. oleracea* and *S. tetrandra* confirmed that the IR regions were more conserved than the LSC/SSC regions (Figs. 3a, b and Table 2). In other respects, the expansion, shrinkage, or difference in boundary region size of LSC/IRs and SSC/IRs is a primary cause of differences in chloroplast genome size^31–33^. Expansions/shrinkage has been reported in many plants, such as in Chinese bayberry^29^, geranium^34^, and green alga^35^. Here, however, no significant IR length variation was detected among the *Spinacia* chloroplast genomes (Fig. S1), which is consistent with *C. quinoa* and *C. album,* which also belong to the family Amaranthaceae^7^. As a large number of variants, including Indels, were located in the LSC/SSC regions (Figs. 3a, b), we thus propose that the length of LSC/SSC was the main contributor to the change in *Spinacia* chloroplast genome size (Fig. S1).

Variations in repeat sequences in the chloroplast are considered important molecular markers and are widely used in plant breeding programs, population genetics, and the identification of commercial cultivars^4,6^. For example, Huang et al. (2015) used chloroplast microsatellite (cpSSR) markers to investigate the genetic relationships between domesticated jujube cultivars and wild relative populations^36^. We identified 46, 46, and 49 repeat sequences (≥ 20 bp) for *S. oleracea*, *S. turkestanica*, and *S. tetrandra* (Table S3). Only 11 polymorphic repeats were obtained between *S. tetrandra* and *S. turkestanica*/*S. oleracea* (both of which shared identical repeats) (Fig. S3a). Similar to the long repeats, both *S. oleracea* and *S. turkestanica* also exhibited identical SSRs (Table S4). Both the long repeats and SSRs were enriched in the intergenic region, which is consistent with previous investigations^4,37^. Gao et al.^38^ found that different types of repeats and their flanking regions could exhibit a high level of variation. Indeed, our study demonstrated that polymorphic long repeats and SSRs could accumulate more variants (Fig. 5f). Actually, variants including indels and SNPs accounted for the polymorphic long repeats or SSRs (Figs. S3b, c), and the variants within the polymorphic repeats could perfectly distinguish *S. oleracea*/*S. turkestanica* and *S. tetrandra* (Fig. S3d). Therefore, the repeats obtained in the study could be used as a valuable resource for studying differences in chloroplast genomes.

A large number of accessions are typically used to reveal the domestication history and evolutionary relationships in nuclear genome analyses, whereas this is seldom the case for the chloroplast genome^2,39^. Here, we used 85 *Spinacia* accessions, including *S. tetrandra*, *S. turkestanica*, and *S. oleracea* individuals, to obtain a comprehensive variant dataset containing 503 SNPs and 83 Indels. On the basis of the variants, remarkably, we found that 13 *S. oleracea* individuals might have crossed with *S. turkestanica*, with the latter acting as the maternal parent (Fig. 4f). Introgression of beneficial traits from wild relatives has contributed to improvement of cultivated crops^40^. For example, quite a few disease resistance loci in cultivated crops were originated from wild relatives, such as powdery mildew resistance loci in watermelon^41^, downy mildew resistance loci in sunflower^42^, and lettuce^43^. Thus, the 13 cultivated spinach varieties that originated through introgression from *S. turkestanica* (Fig. 4f), which is a valuable resource for introducing specific advantageous traits from wild progenitors into cultivated crops. We believed that, apart from the 13 *S. oleracea* accessions, there were still *S. oleracea* accessions crossed with *S. turkestanica* that we could not detect, as *S. turkestanica* served as the paternal parent (Fig. S4a). Importantly, this reminds us that breeders could contribute *Spinacia* genetic exchange and further increase spinach germplasm via reciprocal crossing between cultivated and two wild progenitors (Fig. S4b, c). These results will have a significant meaning for spinach breeding programs, and even provide a breeding reference for other crops.

## Methods

### Plant materials, DNA extraction and sequencing

We used *S. tetrandra*, *S. turkestanica*, and *S. oleracea* from 17S24, 17S31, and inbred line 10S15 for genome resequencing, respectively. Besides the three accessions above, 82 *Spinacia* accessions including seven *S. tetrandra*, 15 *S. turkestanica*, and 60 *S. oleracea* were used for population analysis^13^. The wild relatives were collected from the USA Department of Agriculture (https://npgsweb.ars-grin.gov/), while *S. oleracea* relatives came from the Institute of Vegetables and Flowers (IVF) of the Chinese Academy of Agricultural Sciences (CAAS), Beijing, China (Table S6). The materials used in the study were planted in the field at the IVF of the CAAS in spring 2019. The young leaves from each individual were collected and frozen in liquid nitrogen prior to DNA extraction. High-quality genomic DNA was extracted using a modified cetyltrimethylammonium bromide (CTAB) method^44^. The DNA quality and concentration were measured by electrophoresis on 1% agarose gels and a Thermo Scientific NanoDrop. Then, the Illumina genomic library was constructed and sequenced using a HiSeq 2500 Instrument (Illumina, San Diego, CA, USA) by BioMarker (Beijing, China). Finally, approximately 50 Gb of raw reads were generated for each individual with 150 bp paired-end read lengths. in other respects, the plant materials procured and used in the study comply with China’s guidelines and legislation. All the experiments were carried in accordance with national and international guidelines.

### Chloroplast genome assembly and annotation

Low-quality reads and adapters were filtered using fastp (v0.20.0; parameters ‘-q 20’; https://githup.com/OpenGene/fastp#get-fastp). To identify paired-end reads belonging to the chloroplast genome, we mapped the clean reads against the reference chloroplast genome sequence of *S. oleracea* (GenBank accession number: NC_002202.1)^21^ using bowtie2 (v2.3.5.1; parameters ‘-q’)^45^. The de novo assembly was conducted on mapped paired-end reads using unicycler (v0.4.8) with the default parameters (–min_fasta_length 100–keep 1)^46^. Finally, the chloroplast sequence contigs were ordered and oriented based on the reference chloroplast genome sequences.

The online program GeSeq^47^ (https://chlorobox.mpimp-golm.mpg.de/geseq.html) was used to annotate the *S. tetrandra*, *S. turkestanica*, and *S. oleracea* genomes. Based on a comparison of homologs from previously reported chloroplast genomes of *S. oleracea* in the database and the chloroplast assembly in the study, the three annotation results, specifically the start and stop codons, were further corrected manually. The chloroplast genome maps were constructed using the OGDRAW program^48^.

### Repeat structure and sequence analysis

REPuter^25^ (http://bibiserv.techfak.uni-bielefeld.de/reputer/) was used to find and analyze the sizes and locations of forward, reverse, palindromic, and complementary repeats with a minimal length of 20 bp and a sequence identity greater than 90%^25^.

SSRs (mono-, di-, tri-, tetra-, penta-, and hexanu-cleotide repeats) were identified using MISA (https://webblast.ipk-gatersleben.de/misa/), with thresholds for mono-, di-, tri-, tetra-, penta-, and hexane-nucleotide SSRs of 10, five, five, three, three, and three repeat units, respectively^49^.

### Genetic distance analysis among the *Spinacia* species

To infer the genetic contributions of the two wild progenitors to the cultivated spinach, the genetic divergence between each *S. tetrandra*, *S. turkestanica* and *S. oleracea* was estimated using distmat (http://www.bioinformatics.nl/cgi-bin/emboss/distmat) with the Jukes–Cantor correction for each non-overlapping 1-kb window on the *S. oleracea* chloroplast genome.

### Synonymous (KS) and non-synonymous (KA) substitution rate analysis

Based on the synteny alignments using D-GENIES, homolog genes were identified among the three *Spinacia* species. The yn00 program of PAML v4.9j^50^ was used to estimate K_S_, K_A_, and K_A_/K_S_ values between *S. oleracea* and the two wild relatives. Boxplots were generated with the R software (v3.6.1). The Venn diagram was performed using BMK Cloud platform (http://www.biocloud.net/).

### Phylogenetic analysis

The chloroplast genomes of 14 Amaranthaceae plants were selected for phylogenetic analysis (Table S2), and the *Fagopyrum tataricum* (Polygonaceae) chloroplast genome was used as the outgroup. The IR, LSC, SSC, protein, and complete chloroplast genome sequences were used for phylogenetic analysis using maximum likelihood (ML). First, multiple alignments were performed using MAFFT v7.158b software^51^. As for the protein sequences, 67 genes shared by the 15 species were identified using a custom Python script and then aligned using MAFFT v7.158b. The conserved aligned regions were extracted using Gblock (v0.91b) with parameters ‘–t = p, -b4 = 5, -b5 = h’ to obtain concatenated protein^52^. Finally, each alignment was used to build a maximum likelihood phylogeny using IQ-TREE (v2.1.2; parameters ‘-bb 1000’) with 1000 bootstrap replicates determined by IQ-TREE^53^.

### Variant calling and population genetic analyses

First, two wild relative chloroplast genomes were aligned to the *S. oleracea* chloroplast genome using the Nucmer program^54^ with parameter “-c 80,” followed by the identification of one-to-one alignment blocks using delta-filter with the parameters “-r -q.” Finally, the SNPs and Indels were obtained using show-snp with the parameter “-Clr.” Furthermore, SNPs and Indels within the chloroplast genome and nuclear genome were also detected using large-scale resequencing accessions^13^. Illumina paired-end reads were processed to remove adapters and low-quality sequences using fastp v0.20.1 with the default parameters ^55^. Cleaned reads were mapped to the *S. oleracea* chloroplast genome^21^ using BWA-MEM v0.7.17-r1188 with default parameters^56^, and mapped reads were obtained using SAMtools v1.6–3 with the parameters “-F 12.” As for the variants identified from the nuclear genome, we mapped cleaned reads against the spinach genome^23^ using BWA-MEM v0.7.17-r1188^56^. Variants were called using BCFtools v1.8^57^ and filtered using VCFtools v0.1.16^58^ with the parameters “-maf 0.05 –minQ 30 –max-missing 0.9.” ANNOVAR^59^ was used to annotate the effects of the SNPs and Indels. A neighbor-joining phylogeny was constructed based on the *P* distance matrix calculated by VCF2Dis v1.43 (https://github.com/BGI-shenzhen/VCF2Dis).

## Supplementary Information

Supplementary Figures.

Supplementary Tables.

## Data Availability

The complete chloroplast of the three *Spinacia* have been deposited in the GenBank (accession numbers: MZ516907 for *S. oleracea*, MZ516906 for *S. turkestanica*, and MZ569012 for *S. tetrandra*). The raw sequence data used for assembling chloroplast genomes have been deposited in the Genome Warehouse in BIG Data Center, Beijing Institute of Genomics (BIG), Chinese Academy of Sciences, under accession number CRA005437 that is publicly accessible at https://ngdc.cncb.ac.cn/gsa/.

## References

[1] Yang Y (2021). Coconut genome assembly enables evolutionary analysis of palms and highlights signaling pathways involved in salt tolerance. Commun. Biol..

[2] Sun X (2020). Phased diploid genome assemblies and pan-genomes provide insights into the genetic history of apple domestication. Nat. Genet..

[3] Yang Y (2020). Prickly waterlily and rigid hornwort genomes shed light on early angiosperm evolution. Nat. Plants.

[4] Xue S, Shi T, Luo W, Ni X, Gao ZJ (2019). Comparative analysis of the complete chloroplast genome among *Prunus mume*, P. armeniaca, and P. salicina. Hortic. Res..

[5] Jose CC (2015). A phylogenetic analysis of 34 chloroplast genomes elucidates the relationships between wild and domestic species within the genus citrus. Mol. Biol. Evolut..

[6] Daniell H, Lin CS, Yu M, Chang WJ (2016). Chloroplast genomes: Diversity, evolution, and applications in genetic engineering. Genome Biol..

[7] Hong SY (2017). Complete chloroplast genome sequences and comparative analysis of *Chenopodium quinoa* and C. album. Front. Plant Sci..

[8] Shinozaki K, Ohme M, Tanaka M, Wakasugi T, Hayshida N (1986). The complete nucleotide sequence of the tobacco chloroplast genome. Plant Mol. Biol. Reporter.

[9] Liu L (2018). Chloroplast genome analyses and genomic resource development for epilithic sister genera Oresitrophe and Mukdenia (Saxifragaceae), using genome skimming data. BMC Genom..

[10] Jiang K (2020). Chloroplast genome analysis of two medicinal Coelogyne spp. (Orchidaceae) shed light on the genetic information, comparative genomics, and species identification. Plants.

[11] Dang YY (2014). Complete chloroplast genome sequence of poisonous and medicinal plant *Datura stramonium*: Organizations and implications for genetic engineering. Plos One.

[12] Sajjad A (2017). The complete chloroplast genome of wild rice (*Oryza minuta*) and its comparison to related species. Front. Plant Sci..

[13] She, H. *et al.* The female(XX) and male(YY) genomes provide insights into the sex determination mechanism in spinach. *bioRxiv* (2020).

[14] Group TAP (2016). An update of the angiosperm phylogeny group classification for the orders and families of flowering plants: APG IV. Bot. J. Linn. Soc..

[15] She HB (2021). Identification of a male-specific region (MSR) in Spinacia oleracea. Hortic. Plant J..

[16] Xu C (2015). De novo and comparative transcriptome analysis of cultivated and wild spinach. Sci Rep.

[17] Ribera A, Bai Y, Wolters A, Treuren RV, Kik C (2020). A review on the genetic resources, domestication and breeding history of spinach (*Spinacia oleracea* L.). Euphytica.

[18] Ribera A (2020). On the origin and dispersal of cultivated spinach (*Spinacia oleracea* L.). Genet. Resources Crop Evolut..

[19] Treuren R (2020). Acquisition and regeneration of Spinacia turkestanica Iljin and S. tetrandra Steven ex M. Bieb. to improve a spinach gene bank collection. Gen. Resources Crop Evolut..

[20] Handke S, Seehaus H, Radies M (2000). Detection of a linkage of the four dominant mildew resistance genes 'M1M2M3M4' in spinach from the wildtype Spinacia turkestanica. Gartenbauwissenschaft.

[21] Schmitz-Linneweber C (2001). The plastid chromosome of spinach (Spinacia oleracea): complete nucleotide sequence and gene organization. Plant Mol. Biol. Report..

[22] Zou C (2017). A high-quality genome assembly of quinoa provides insights into the molecular basis of salt bladder-based salinity tolerance and the exceptional nutritional value. Cell Res..

[23] Xu C (2017). Draft genome of spinach and transcriptome diversity of 120 Spinacia accessions. Nat. Commun..

[24] Ni ZX, Zhou PY, Xin Y, Xu M, Xu LA (2021). Parent-offspring variation transmission in full-sib families revealed predominantly paternal inheritance of chloroplast DNA in *Pinus massoniana* (Pinaceae). Tree Genet. Genom..

[25] Kurtz S (2001). REPuter: the manifold applications of repeat analysis on a genomic scale. Nucleic Acids Res..

[26] Henry RJ (2005). Plant diversity and evolution: Genotypic and phenotypic variation in higher plants.

[27] Jansen RK, Cai Z, Raubeson LA, Daniell H, Depamphilis CW, Leebens-Mack J, Müller KF, Guisinger-Bellian M, Haberle RC, Hansen AK, Chumley TW (2008). Analysis of 81 genes from 64 plastid genomes resolves relationships in angiosperms and identifies genome-scale evolutionary patterns. Proc. Natl. Acad. Sci..

[28] Li R, Ma PF, Wen J, Yi TS (2013). Complete sequencing of five araliaceae chloroplast genomes and the phylogenetic implications. Plos One.

[29] Liu LX (2017). The complete chloroplast genome of chinese bayberry (Morella rubra, Myricaceae): Implications for understanding the evolution of fagales. Front. Plant Sci..

[30] Lu RS, Li P, Qiu YX (2016). The complete chloroplast genomes of three cardiocrinum (Liliaceae) species: Comparative genomic and phylogenetic analyses. Front. Plant Sci..

[31] Hui C, Li J, Hong Z, Cai B, Lin M (2017). The complete chloroplast genome sequence of strawberry (Fragaria ×ananassa Duch.) and comparison with related species of Rosaceae. PeerJ..

[32] Ni L, Zhao Z, Gaawe D, Mi M, Chen S (2016). The complete chloroplast genome of Ye-Xing-Ba (*Scrophularia dentata*; *Scrophulariaceae*), an Alpine Tibetan Herb. PLOS ONE.

[33] Wang RJ (2008). Dynamics and evolution of the inverted repeat-large single copy junctions in the chloroplast genomes of monocots. BMC Evol. Biol..

[34] Chumley TW (2006). The complete chloroplast genome sequence of pelargonium × hortorum: Organization and evolution of the largest and most highly rearranged chloroplast genome of land plants. Mol. Biol Evol.

[35] Qra B (2021). The extremely large chloroplast genome of the green alga Haematococcus pluvialis: Genome structure, and comparative analysis. Algal Research.

[36] Jian H (2015). Development of chloroplast microsatellite markers and analysis of chloroplast diversity in Chinese Jujube (*Ziziphus jujuba* Mill.) and Wild Jujube (*Ziziphus acidojujuba* Mill.). PLoS ONE.

[37] Yang Y (2016). Comparative analysis of the complete chloroplast genomes of five quercus species. Front. Plant Sci..

[38] Gao L (2019). Evolution of Oryza chloroplast genomes promoted adaptation to diverse ecological habitats. Commun. Biol..

[39] Wei T, Treuren RV, Liu X, Zhang Z, Liu H (2021). Whole-genome resequencing of 445 Lactuca accessions reveals the domestication history of cultivated lettuce. Nat. Genet..

[40] Lin T (2014). Genomic analyses provide insights into the history of tomato breeding %J Nature Genetics. Nat. Genet..

[41] Guo S (2019). Resequencing of 414 cultivated and wild watermelon accessions identifies selection for fruit quality traits. Nat. Genetic.

[42] Hübner S, Bercovich N, Todesco M, Mandel JR, Rieseberg LH (2019). Sunflower pan-genome analysis shows that hybridization altered gene content and disease resistance. Nat. Plants.

[43] Wei T (2021). Whole-genome resequencing of 445 Lactuca accessions reveals the domestication history of cultivated lettuce. Nat. Genet..

[44] Allen (2006). A modified protocol for rapid DNA isolation from plant tissues using cetyltrimethylammonium bromide. Nat. Protoc..

[45] Langmead B, Salzberg SL (2012). Fast gapped-read alignment with Bowtie. Nat. Methods.

[46] Wick RR, Judd LM, Gorrie CL, Holt KE (2017). Unicycler: Resolving bacterial genome assemblies from short and long sequencing reads. PLoS Comput. Biol..

[47] Tillich M (2017). GeSeq: versatile and accurate annotation of organelle genomes. Nucleic Acids Res.

[48] Greiner S, Lehwark P, Bock R (2019). OrganellarGenomeDRAW (OGDRAW) version 1.3.1: Expanded toolkit for the graphical visualization of organellar genomes %J Nuclc Acids Research. Nuclc Acids Res..

[49] Beier S, Thiel T, Münch T, Scholz U, Mascher M (2017). MISA-web: A web server for microsatellite prediction. Bioinformatics.

[50] Yang ZH (2007). PAML 4: Phylogenetic analysis by maximum likelihood. Mol. Biol. Evol..

[51] Katoh K, Kuma K-I, Toh H, Miyata T (2005). MAFFT version 5: Improvement in accuracy of multiple sequence alignment. Nucleic Acids Res..

[52] Gerard T, Jose C (2007). Improvement of phylogenies after removing divergent and ambiguously aligned blocks from protein sequence alignments. Syst. Biol..

[53] Lam-Tung N, Schmidt HA, Arndt VH, Quang MB, & Evolution (2015). IQ-TREE: A fast and effective stochastic algorithm for estimating maximum-likelihood phylogenies. Mol. Biol. Evolut..

[54] Kurtz S, Phillippy A, Delcher AL, Smoot M (2004). Versatile and open software for comparing large genomes. Genome Biol..

[55] Chen S, Zhou Y, Chen Y, Gu J (2018). fastp: an ultra-fast all-in-one FASTQ preprocessor. Bioinformatics.

[56] Li, H. Aligning sequence reads, clone sequences and assembly contigs with BWA-MEM. (2013) arXiv [q-bioGN], http://arxiv.org/abs/1303.3997.

[57] Li H (2011). A statistical framework for SNP calling, mutation discovery, association mapping and population genetical parameter estimation from sequencing data. Bioinformatics.

[58] Danecek P (2011). The variant call format and VCFtools. Bioinformatics.

[59] Kai W, Li M, Hakon H (2010). ANNOVAR: functional annotation of genetic variants from high-throughput sequencing data. Nucleic Acids Res..

