# Supplementary material for: Comparative chloroplast genome analyses of cultivated spinach and two wild progenitors shed light on the phylogenetic relationships and variation
Source: Sci Rep. 2022 Jan 17;12:856. doi: 10.1038/s41598-022-04918-4 (PMC8763918; doi:10.1038/s41598-022-04918-4)
Supplement: Supplementary file 1 — Supplementary Figures. [file 41598_2022_4918_MOESM1_ESM.pdf]

Comparative chloroplast genome analyses of cultivated spinach and two wild progenitors shed light on the phylogenetic relationships and variation

Hongbing She, Zhiyuan Liu, Zhaosheng Xu, Helong Zhang, Feng Cheng, Jian Wu, Xiaowu Wang, Wei Qian*

Institute of Vegetables and Flowers, Chinese Academy of Agricultural Sciences, Beijing 100081, China

*** Correspondence:**

Wei Qian, qianwei@caas.cn, Tel: +86-010-62194559

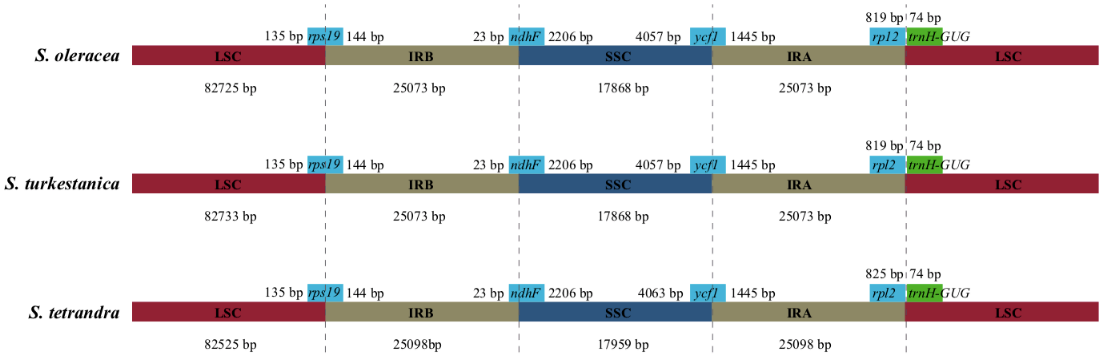

Fig. S1 Comparison of the borders of the LSC, SSC, and IR regions of the chloroplast genomes in the three *Spinacia* species.

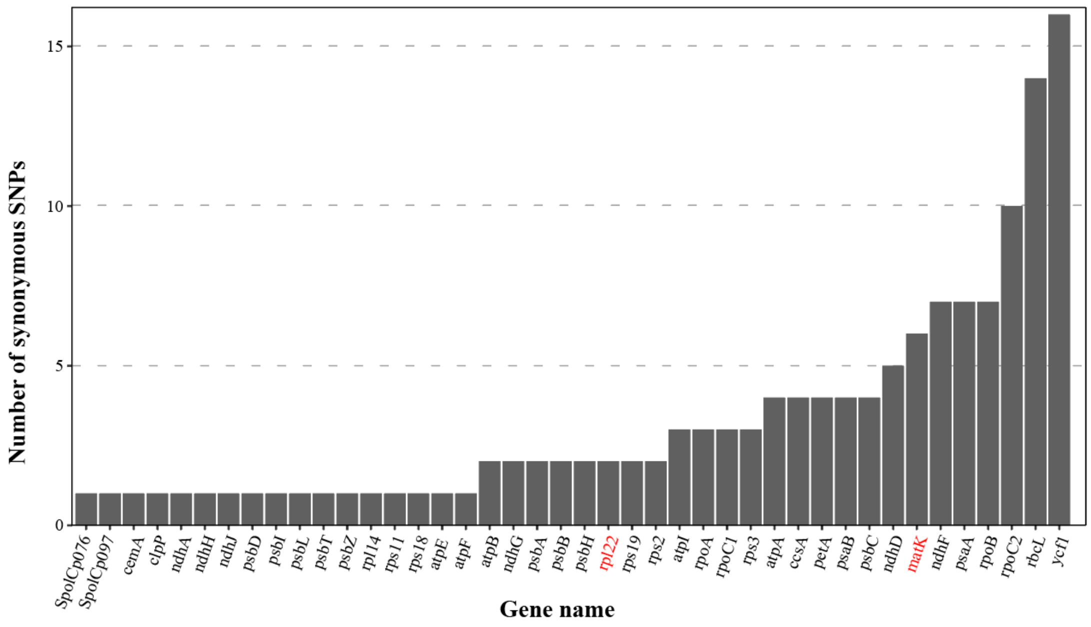

Fig. S2 Distribution of 92 synonymous SNPs on genes. One and two Indels are situated in *rpl22* and *matK*, respectively, and are represented by red, resulting in frameshifts.

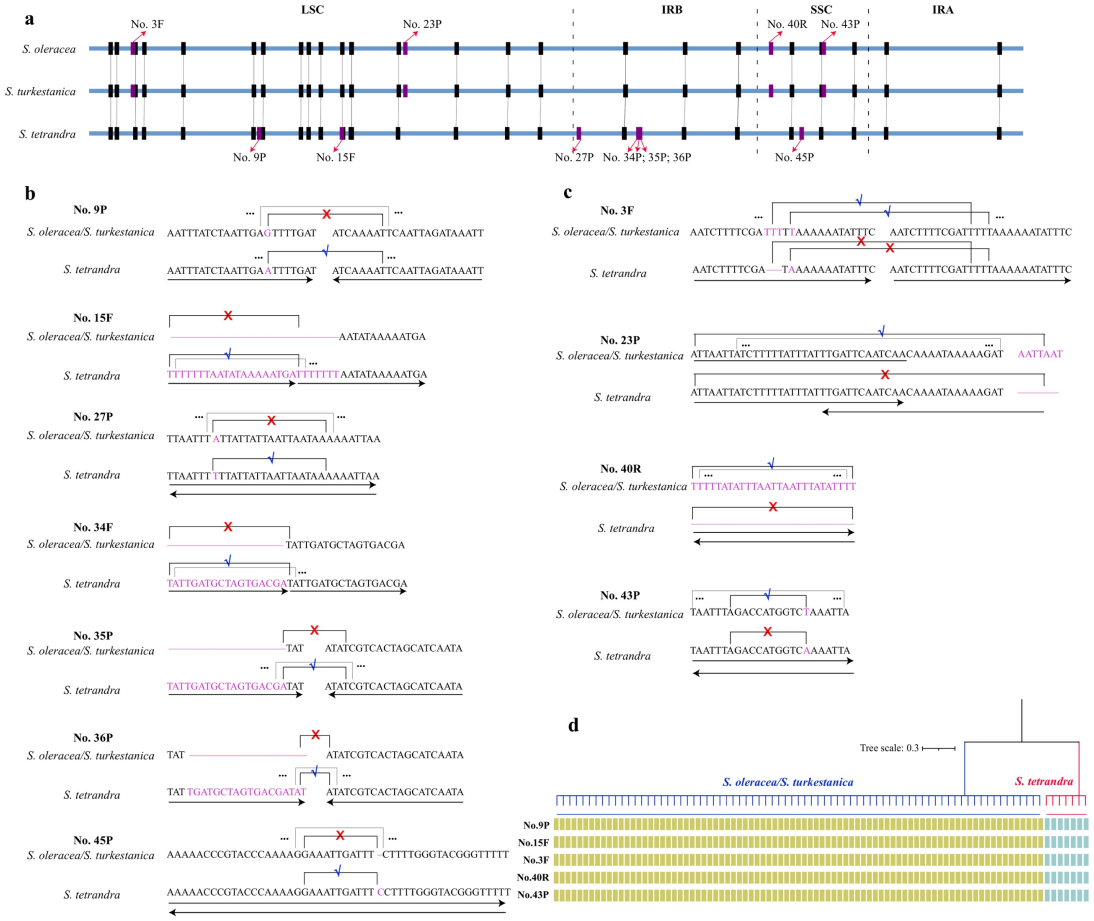

Fig. S3 Analysis of long repeat sequences in the three *Spinacia* chloroplast genomes. **(a)** Distribution of long repeat sequences. The rectangles in black and purple indicate non-polymorphic and polymorphic long repeats, respectively. Only a single repeat from a pair of long repeats was drawn. The name of the polymorphic long repeat is indicated by a red arrow. The letters “P”, “F’, and “R” within the name represent palindromic, forward, and reverse repeats, respectively. Sequences of **(b)** seven S. tetrandra-specific long repeats and **(c)** four *S. oleracea*-specific or *S. turkestanica*-specific long repeats. The symbol “√” indicates that the sequence was a repeat, while “X” indicates that it was not. The two arrows represent a pair of long repeats. **(d)** A phylogenetic tree was constructed using 82 *Spinacia* accessions based on the five variants identified from the

polymorphic long repeats. Yellow rectangles indicate that the homozygous genotype in the accession was consistent with the *S. oleracea* chloroplast genome, whereas blue rectangles indicate that the homozygous genotype differed from the *S. oleracea* chloroplast genome.

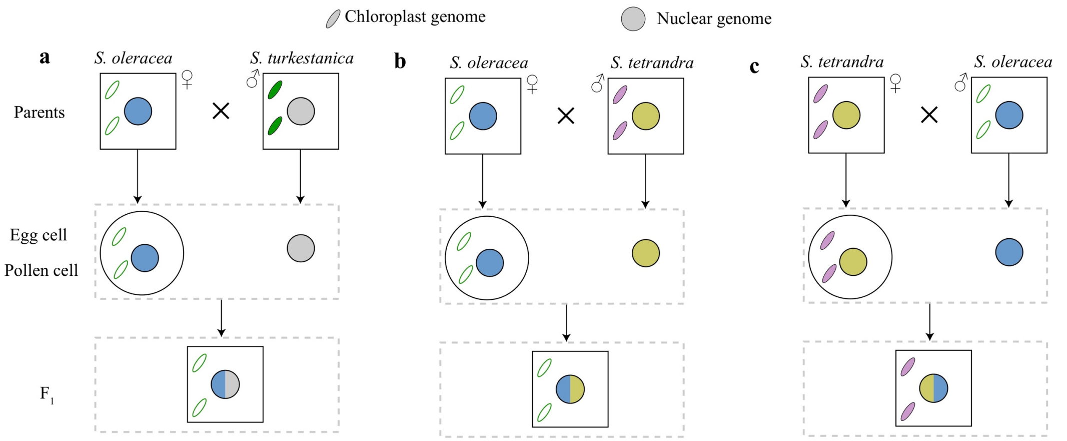

Fig. S4 Summarized inheritance patterns of chloroplast in spinach. **a–c** indicate multiple approaches to creating new spinach germplasm.
